# Hand eczema and skin complaints in particulate matter-exposed occupations - firefighters, chimney sweepers, and ferrosilicon smelter workers in Norway

**DOI:** 10.1186/s12995-024-00407-8

**Published:** 2024-03-14

**Authors:** Krister Aune Teigen, Anje Christina Höper, Solveig Føreland, Merete Åse Eggesbø, Marit Nøst Hegseth

**Affiliations:** 1 Department of Occupational and Environmental Medicine, University Hospital of North Norway, Tromsø, Norway; 2 Department of Community Medicine, UiT The Arctic University of Norway, Tromsø, Norway; 3 Department of Occupational Medicine, Haukeland University Hospital, Bergen, Norway; 4 Department of Environmental Medicine, Norwegian Institute of Public Health, Oslo, Norway; 5 Department of Clinical and Molecular Medicine, NTNU, Trondheim, Norway

**Keywords:** Occupational dermatitis, Occupational medicine, Occupational exposure, Particle, Nanoparticles, Skin complaints

## Abstract

**Background:**

The objective was to investigate self-reported hand eczema, and skin complaints at other skin locations among workers exposed to particulate matter, especially ultrafine particles.

**Method:**

We conducted a cross-sectional study on workers from one ferro-silicon smelter plant, eight chimney sweeper stations and one firefighter station across Norway. Participants answered an extended version of the Nordic Occupational Skin Questionnaire (NOSQ-2022), with additional questions about whole-body skin complaints and visible dust deposition. Results are presented as descriptive data using firefighters as reference group. Odds ratio (OR) was calculated using logistic regression on lifetime prevalence of hand eczema adjusted for potential confounders and mediators. P-values were calculated using likelihood ratio test against the crude OR.

**Results:**

A total of 186 participants answered the questionnaire: 74 chimney sweepers, 52 firefighters and 60 smelter workers. Participation rate was 95.0, 94.5 and 63.6%, respectively. Lifetime prevalence of hand eczema was 9.5, 9.6, and 28.3%, respectively. The point prevalence of hand eczema was 1.4, 1.9 and 10.0%, respectively. We estimated OR for lifetime hand eczema in smelter workers to 4.36 [95% CI: 1.31–14.43, *p* = 0.016] and for lifetime skin complaints in other locations to 2.25 [95% CI: 0.98–5.18, *p* = 0.058]. The lifetime prevalence of skin complaints at other locations was 18.9, 23.1 and 40.0%, respectively. The point prevalence was 14.9, 9.6 and 16.7%, respectively. These estimates were not statistically significant but indicates that smelter workers have more skin complaints also at other locations.

**Conclusion:**

This study reports a more than four-fold increased risk of hand eczema in smelter workers, and possibly a higher risk of skin complaints in other body locations, compared to the other occupations. Longitudinal studies with larger population are needed to verify the marked increased risk of eczema among smelters and establish causation.

## Introduction

Occupational dermatoses are among the most reported occupational diseases and contact eczema accounts for 80–90% of all reported occupational dermatoses in the European Union [1, 2]. Contact eczema is caused by underlying skin inflammation due to external exposure to allergens or irritants. The mechanism of irritant contact eczema involves repeated exposure to factors such as wet work, detergents, oils, mechanical friction, heat, cold, sweat, and dust. The skin can tolerate a certain level of exposure to irritants before its regenerative capacity is surpassed, and eczema develops [1, 3]. The most crucial internal risk factor for contact eczema is atopic eczema [4], whereas wet work is the most common external risk factor [5]. Wet work is defined as contact with water for more than 2 h per day, hand washing over 20 times per day, or using airtight gloves for more than 2 h per day [5]. Clinical distinction between allergic or irritant contact dermatitis is almost impossible and warrants testing with standardized patch-testing. In up to 80% of cases, contact eczema occurs on the hands, wrists, or forearms, which are typical locations for contact with external exposure, and is often referred to as hand eczema [4]. Studies have shown that lifestyle factors may increase the risk of hand eczema, for instance obesity [6, 7]. Some population studies have also found a higher risk in tobacco smokers, but others have not [7–9].

A population-based study in Norway reported a lifetime prevalence of contact eczema of 8.4 and 13.8% in men and women, respectively, and a lifetime prevalence of occupational hand eczema of 4.8% [7, 10]. Work-related hand eczema is associated with occupations in health care, social work, and occupational cleaning in women, and industrial occupations and farming in men [7]. In a systematic review of hand eczema, primarily based on studies from Nordic countries, the pooled lifetime and point prevalence for both sexes were 15.6 and 4.0%, respectively [10].

Hand eczema has a significant impact on workability, as well as physical, social, and mental health. Substantial financial consequences apply to the patient, workplace, and society. Annual costs per patient with hand eczema are estimated to be between €1311 and €9792 [11]. Consequently, preventive interventions may provide substantial personal and economic benefits.

There are approximately 3700 firefighters [12], 800 chimney sweepers [13], and 7000 people working in the smelter industry in Norway (retrieved and summed up from all known smelters websites in Norway). By experience, workers in these occupations are exposed to multiple risk factors for hand eczema such as wet work, different chemicals, particulate matter containing different sized particles, including ultrafine particles (UFP), and more.

UFP forms during combustion, such as in a diesel engine, during fires, and during metallurgical processes [14–18]. UFP are sized between 1 and 100 nanometres (nm) and are often referred to as PM0.1 [19]. UFP differ from PM10 and PM2.5 with regards to aerodynamic properties resulting in different mobility pattens. Also, UFP have a large surface area relative to their small mass and may therefore be efficient carriers of chemical compounds such as metals etc. [20]. Several metals are known to potentially cause contact dermatitis, such as nickel, chromium, cobalt etc. [21–24].

Dermal exposure to small particulate matter such as UFP may impose a risk for adverse health effects. A comparative study from 2021 investigated the effects of PM2.5 exposure and skin barrier function and reported that PM2.5 exposure compromised skin barrier function and resulted in increased transepidermal water loss [25]. Furthermore, smaller fractions (UPF/PM0.1) may penetrate healthy skin through the hair follicles and sweat glands [26]. Damaged skin, such as atopic eczema, may subsequently increase the skin permeability of UFP 4–100 times [27, 28]. Particulate matter containing metals may also act as skin allergens, causing eczema [23, 24], which may cause systemic uptake of particles [26, 29]. Skin barrier dysfunction, such as eczema, on any body part is therefore relevant because UFP can be deposited on different body parts and gain access to the skin through clothing and clothing openings. This is relevant n occupations exposed to particulate matter [30, 31].

Workers in the smelter industry comprise diverse occupational groups, and their exposure depends on the type of product manufactured and the type of work they perform. The smelter plant in this study produced ferrosilicon. Particulate matter of various sizes (PM10, PM2.5, UFP (PM0.1)) are generated during smelting, handling of raw materials, refining, and crushing of the product, and are primarily silica (SiO) and amorphous silica (SiO2). They also contain other volatile organic compounds such as benzene and polycyclic aromatic hydrocarbons (PAH) as well as various metals absorbed on the particulate matter [17, 32].

During firefighting, firefighters are exposed to asphyxiants, irritants and other volatile organic compounds, polycyclic aromatic hydrocarbons (PAH), and particulate matter of various sizes (PM10, PM2.5, UFP (PM0.1)) [14–16, 33]. Firefighters’ exposure to fire smoke will depend on fire scenario (ventilation, fuel), type, frequency and duration of fires they attend to, use of personal protective equipment etc. [34–38]..

Chimney sweepers’ work environment is associated with pyrogenic chemicals including volatile and semi-volatile organic compounds, such as benzene, PAHs, PM etc. The particulate matter fraction is mainly larger than PM0.1 [39]. There is limited knowledge about ultrafine particle exposure in chimney sweepers. Our study group conducted a study with measurements of ultrafine particles. We found UFP counts ranging from time weighted average (TWA) 2.0 × 10^3^ − 7.1 × 10^4^ particles/cm^3^, where sweeping at a waste burning facility generated the highest particle count [40] Chimney sweeping at other locations only generated small UFP counts.

All these numbers are time weighted averages over a work period of eight hours and some tasks yielded UFP-count above and near the proposed nano reference limit time weighted average value of 10 × 10^4^ particles/cm^3^.

In this study we investigate the prevalence of hand eczema and skin complaints among workers in occupations with known particle matter exposure, and especially UFP exposure. The objective was to assess self-reported lifetime- and point-prevalence of hand eczema, as well as skin complaints at other locations, among smelter workers, firefighters and chimney sweepers. To our knowledge, no previous studies have investigated hand eczema and skin complaints among these professions.

## Materials and methods

## Study design

A quantitative, descriptive, cross-sectional study was chosen to fulfil the aim of the study. The study is part of a larger project, SCINDEEP, which focuses on airborne UFP exposure among smelter-workers, firefighters and chimney sweepers [41]. We invited 80 chimney sweepers across eight locations in Norway, 55 firefighters from one fire station and 99 smelter workers from one ferro-silica smelter plant. The eight locations chosen for chimney sweepers were larger cities, due to larger number of employees. The ferro silicon plant and firefighters were recruited based on established contacts from previous studies and the proximity to study group members. We contacted managers at each location and if they were positive to the study, we organised physical and digital information meetings with the administration and workers. All workers with particulate matter exposure at least one hour per day, or working as a firefighter, were eligible for participation. For smelter workers and chimney sweepers, this meant working within the furnace or with active chimney sweeping for at least 1 h per day. All participants were asked to fill out a daily log scheme to ensure this eligibility criterium was met. Chimney sweepers were recruited from November 2021 to June 2022, firefighters from December to May 2021, and smelter workers from August 2018 to December 2019. The participation rate to the questionaries was 94.5% among firefighters, 95.0% among chimney sweepers, and 61.6% among smelter workers.

### Outcome

**Fig. 1:**
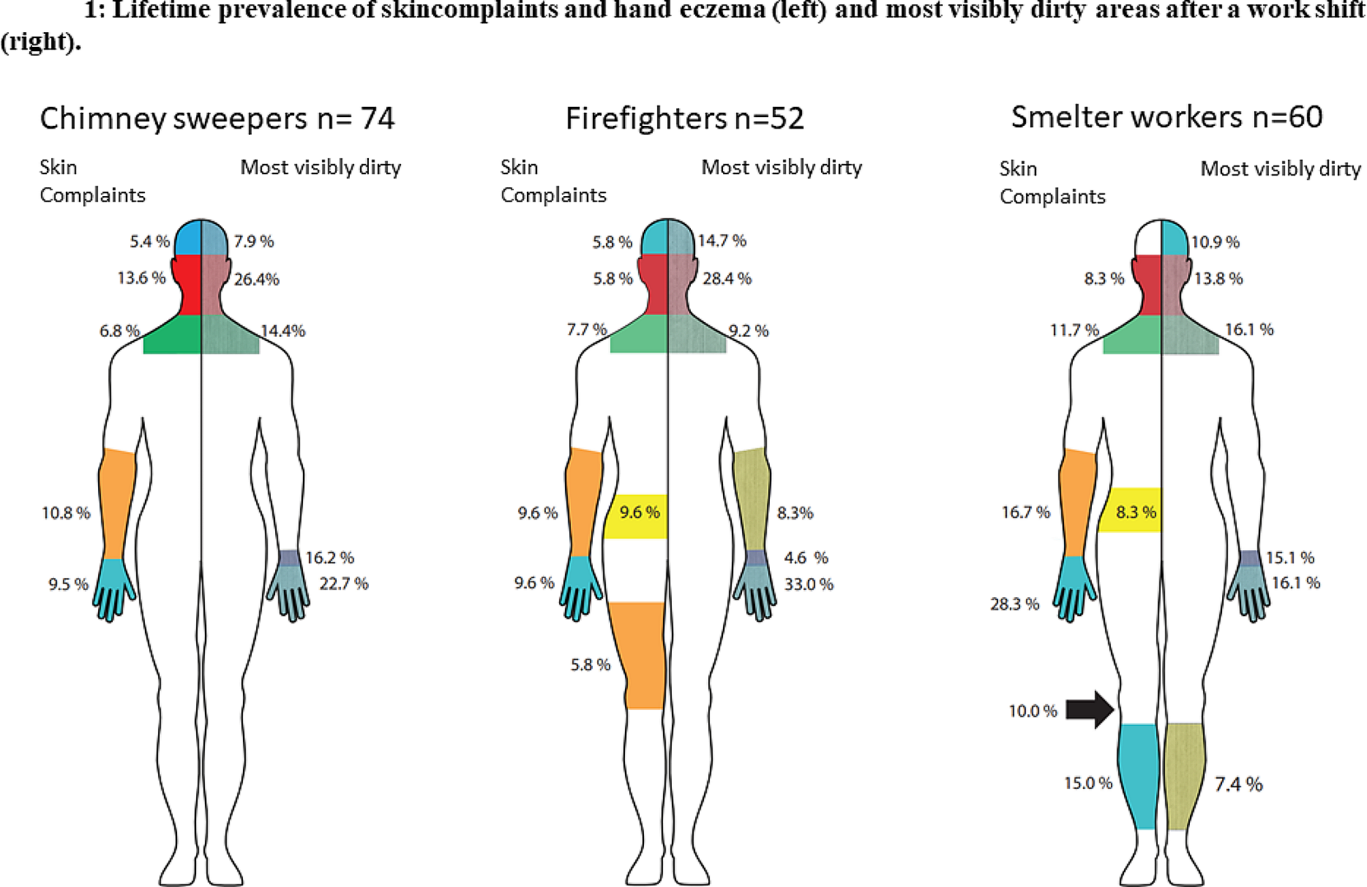
Left side of mannequin: Lifetime prevalence of skin complaints and hand eczema. Colours mark the different locations together with the respective percentages of participant reporting skin complaints and hand eczema. Right side of mannequin: Locations where participants marked where they are most visibly dirty after work-shift/firefighting. Colours mark the different locations together with the respective percentages reporting visible dirt at the location

Participants were asked to answer an extended version of the standardised Northern Occupational Skin Questionnaire-2002 (NOSQ-2002) [42]. The NOSQ-2002 contains questions regarding work-related hand eczema, urticaria, atopic history, allergies, and occupational- and off-duty exposure. The extension included questions regarding skin complaints on other parts of the body, medical history and smoking status. The participants were asked to draw the location of hand eczema, and skin problems in other areas, on a mannequin (Fig. 1).

Participants with current skin problems underwent clinical skin examinations by Krister Aune Teigen, junior doctor in occupational medicine. The main purpose was to secure quality of the answers in the questionnaire and give medical advice where required. Participants with severe complaints were referred to their general practitioner for further follow-up. Images of visible efflorescence were captured and stored in a secure research server at the UNN along with questionnaire data. Some participants were not examined owing to lack of consent, some did not have any skin problems at the time of sampling, and others were not available for examination. Out of the 33 with reported hand eczema and/or skin complaints at other locations at the time of sampling, 22 workers underwent clinical examination.

### Particle exposure

Participants were presented a mannequin in the questionnaire and asked to mark the locations where they had most visible particle deposition and grade it from 1 to 6, with 1 being the most contaminated and 6 being the least contaminated. Furthermore, exposed participants filed a daily log scheme containing questions on amount of time exposed to particles, type of tasks conducted, and the use of respiratory protective equipment to better estimate their daily particle exposure. The number of hours of daily particle exposure was reported as daily average over four consecutive workdays. Four days were selected to provide an as equal amount of work hours as possible between the groups, since the smelter workers worked only for four days consecutively.

### Potential confounders and mediators

Confounding factors and mediators were identified by drawing a directed acyclic graph (DAG) using information obtained from the questionnaire [43]. With occupation as the exposure and hand eczema/skin complaints as the outcome, the potential confounding factors identified were atopic dermatitis, other skin disease which compromises the skins integrity, sex, tobacco smoking and education. The DAG also identified potential mediators: exposure to heat, cold, chemicals, protective glove use, duration and concentration of exposure and type of UFP. Showering and handwashing frequency were identified as colliders.

### Statistical analysis

We characterised the workers according to occupation, sex, education level, snus, smoking, atopic eczema BMI and prevalence of hand eczema and skin complaints (Table 1). A logistic regression model was used to analyse the association. We chose firefighters as a reference because they were the occupational group assumed to have the lowest UFP exposure. The firefighters in our study had only occasional callouts to fires and were thereby not exposed to ultrafine particles from fires daily.

Using likelihood ratio test, we compared models with and without the potential confounding factors and mediators. Workers’ BMI, snus, smoking and education had no confounding effects on the estimates, whereas atopic eczema and sex did, and were therefore kept in the final model. We tested mediators similarly with and without the mediator.

STATA/MP 16.1 (StataCorp LLC, TX, USA) was used for statistical analysis.

## Results

In total, 189 participants answered the questionnaire. Two chimney sweepers and one smelter worker were removed from the study set because of missing answers on crucial questions on hand-eczema/skin complaints. The final study subset contained 186 participants: 74 chimney sweepers, 52 firefighters and 60 smelter workers.

Table 1 show the characteristics of the study population. There was an overweight of men (91.4%) and the majority were educated at elementary/high school level (86%). A substantial number of participants used snus, and the percentage of smokers was highest in smelter workers. The mean BMI in all groups was in the “overweight” category. Firefighter reported more years working in the occupation.

**Table 1:** Characteristics of study population

Occupation	Chimneysweeper			Firefighter			Smelter			Total		
N	74			52			60			186		
Gender	n		%	n		%	n		%	n		%
Man	62		83.8%	51		98.1%	57		95.0%	170		91.4%
Woman	12		16.2%	1		1.9%	3		5.0%	16		8.6%
**Education**												
Elementary/highschool	67		90.5%	41		78.8%	52		86.6%	160		86.0%
College/university (min 2 year)	7		9.5%	11		21.2%	8		13.3%	26		14.0%
**Tobacco**												
Smoking	11		14.9%	6		11.5%	17		28.3%	34		18.3%
Snus	34		46.0%	17		32.7%	24		40.0%	75		40.3%
	Mean	Median	Range	Mean	Median	Range	Mean	Median	Range	Mean	Median	Range
**BMI in kg/m2** (9 missing)	27.0	26.5	(19.6–46.3)	26.3	25.9	(22.4–33.2)	29.3	28.3	(19.7–55.3)	27.6	26.6	(19.6-55.3)
**Age in years**	36.8	34	(19–60)	41.9	39.5	(25–55)	37.6	35	(18–71)	38.3	38	(18-71)
**Years in occupation** (1 missing)	10.8	8	(0.2–35)	15.0	13	(1–32)	10.3	6	(0.2–37)	11.6	10	(0.2-37)
**Work–hours per week** (7 missing)	37.6	37.5	(30–40)	42.2	42	(24–68)	35.0	37.5	(15–40)	38.0	37.5	(15-68)

A Total of 36.6% (68 participants) reported hand eczema and/or skin complaints at other locations (Table 2). 11 of the 68 participants reported both hand eczema and skin complaints combined where 2 were chimney sweepers, 0 were firefighter and 9 were smelter workers.

**Table 2:** Skin complaints within the occupational groups

Occupation	Chimneysweeper		Firefighter		Smelter		Total		χ^2^
N	74		52		60		186		Fisher
**Childhood eczema (Atopic dermatitis)**	7	9.5%	3	5.8%	6	10.0%	16	8.6%	0.659
**Participants with hand eczema and/or skin complaints in total**	**19**	**25.7%**	**17**	**32.7%**	**32**	**53.3%**	**68**	**36.6%**	0.004
Lifetime prevalence hand eczema	7	9.5%	5	9.6%	17	28.3%	29	15.6%	0.004
Lifetime prevalence skin complaints other locations	14	18.9%	12	23.1%	24	40.0%	50	26.9%	0.020
Point prevalence hand eczema	1	1.4%	1	1.9%	6	10.0%	8	4.3%	0.732
Point prevalence skin complaints other locations	11	14.9%	5	9.6%	10	16.7%	26	14.0%	0.781

### Hand Eczema

The lifetime prevalence is reported in Table 2. The 12 months prevalence of skin symptoms from hands, wrists, or forearm was 25.7% for chimney sweepers, 38.5% for firefighters and 56.7% for smelter workers. The point-prevalence of hand eczema was 1.4 1.9 and 10.0% respectively. Chimney sweepers reported a mean of 2.6 symptoms (range: 0–5), firefighters 2.8 (range:0–5) and smelter workers 3.4 (range: 0–10). The most reported symptoms were cracks in the skin (29.3%), dry and flaky skin (22,5%) and itch (17,2%). 29.4% of smelter workers reported having hand eczema “(almost) all the time” compared to 0% of chimney sweepers and firefighters.

25% of chimney sweepers, 12,5% of firefighters and 47% of the smelter workers with hand eczema were working in their current occupation at the onset of hand eczema. Among the participants with hand eczema, 20% of chimney sweepers, 40% of the firefighters and 29.4% of smelter workers reported worsening factors at work. The most common worsening factor among smelter workers was contact with oils/chemicals (80.0%), followed by dust exposure (60%). Two chimney sweepers answered sweat/moisture and contact with cleaning wipes, respectively, while one firefighter answered “contact with soap” as the worsening factor at work. Improvement of hand eczema away from work was reported by 58.8% of the smelter workers, 25.0% of the chimney sweepers and 12.5% of firefighters.

Two of the seventeen smelter workers with hand eczema reported sick leave because of hand eczema.

### Skin complaints at other locations

Lifetime prevalence of skin complaints at other locations was highest among smelter workers and lowest in chimney sweepers (Table 2). Point prevalence in chimney sweepers, firefighters and smelter workers was, 14.9, 9.6 and 16.7%, respectively. Itching was the most reported complaint (88.9%), followed by dry and flaky skin (44.4%). Among participants having skin complaints, 35.7% of chimney sweepers, 16.6% of firefighters and 58.3% of smelter workers reported worsening factors at work. Dust exposure was the most reported worsening factor among the smelter workers (72.7%) and chimney sweepers (75%), while a single firefighter reported cold exposure as worsening factor.

Improvement in skin complaints away from work was reported by 42.9% of the chimney sweepers, 16.5% of firefighters and 58.3% of smelter workers with skin complaints.

Smelter workers differentiated from the two other groups where they reported skin complaints from behind the knee (10%) and on shin areas (15%) (Fig. 1). 33% and 50%, respectively, reported that dust exposure worsened the symptoms.

### Clinical skin examination

Clinical skin examination was conducted on 22 smelter workers, 5 firefighters and 3 chimney sweepers. Symptoms and signs in firefighters and chimney sweepers were primarily minor. Smelter workers reported more severe symptoms, such as itching, bloody skin, and worsening skin problems, after contact with large amounts of dust or handling raw materials. In addition, smelter workers had more visible signs, such as macules and papules on hands with minor wounds, dry and flaky skin, and secondary efflorescence, such as excoriation.

Among the 17 smelter workers with hand eczema, three participants were considered to have possible classical occupational contact eczema based on anamnesis and clinical skin examination. The patients were referred to a dermatologist and a family doctor. One participant from smelter workers was given a standardized patch test with addition of dust from the smelter plant. The test for dust was negative.

### Particle deposition on skin

Smelter workers marked a significantly higher number of locations where they were visibly dirty after a work-shift with a mean of 5.1 locations. Firefighters and chimney sweepers marked a mean of 2.0 and 2.8 locations respectively (Fig. 1, right side of mannequins). We also observed the smelter workers had more visible deposition of black dust on their skin compared to the chimney sweepers and firefighters. We conducted tape sampling to a collateral study involving electron microscopy from the workers’ skin, which revealed substantial amounts of dust on smelter workers (article in review). Respondents across all industries ranked their hands and wrists as the most visibly dirty, followed by their faces. These locations corresponded to where workers reported having skin complaints (Fig. 1, left sides of mannequins).

### Exposures

The occupational groups reported similar frequencies of hand washing, weekly showering, and hourly wet work (contact with water) (Table 3). Smelter workers reported more hours of daily heat exposure, daily particulate matter exposure, and more protective glove usage than firefighters and chimney sweepers (mean hours per day: 4.9 vs. 1.6 and 3.2). Smelter workers reported using leather/cotton/fabric gloves as the primary glove, whereas 74% of chimney sweepers reported using airtight gloves, including rubber and vinyl.

**Table 3:** Daily work exposures across occupational groups

Exposures/Occupation	Chimney sweeper	Firefighter	Smelter
N	74	52	60
Daily handwashing			
< 20 times per day	96%	94%	98%
> 20 times/day	4%	6%	2%
**Weekly showering**			
1–6 times per week	46%	52%	52%
7–9 times per week	30%	38%	35%
>10 times/week	24%	10%	13%
**Contact with water**			
<2 h per day	99%	96%	100%
>2 h per day	1%	4%	0%
**Heat exposure**			
0.5–2 h/day	8%	13%	15%
> 2 h/day	7%	0%	57%
**Cold exposure**			
0.5–2 h/day	11%	22%	18%
> 2 h/day	14%	4%	16%
**Use of chemicals**			
0.5–2 h/day	13%	59%	22%
>2 h per day	0%	2%	5%
**Particulate matter exposure >2 h/day**	55.4%	0%	75%
Mean hours per day (range)	2.3 (0–5)	n/a*	4.1 (0–9.3)
**Protective glove use**			
Mean hours per day (range, missing)	3.2 (1-7, 11)	1.6 (1-5, 25)	4.9 (1-10, 18)
**Type of glove**			
Rubber, pvc,nitrile, latex, PE	74.3% (55)	78.9% (41)	8.33% (5)
Leather	40.5% (30)	51.9% (27)	55% (33)
Fabric/cotton	33.7% (25)	23.1% (12)	61.7% (37)

Missing average across exposure categories: 1.07% (range: 0–6)

*Firefighting occurs at different times. Daily particle exposure from fire smoke not avaliable

### Association between occupation, exposure, and skin complaints

The crude and adjusted ORs for lifetime hand eczema and skin complaints at other locations are presented in Table 4. The crude OR of lifetime prevalence of hand eczema was almost four-fold higher in smelter workers than firefighters. It increased to over four-fold OR in the adjusted model. There was a similar pattern in lifetime skin complaints in other locations, although not statistically significant.

Mediator analysis with significance testing was conducted for each exposure factor in Table 4 and none of the factors were found to influence the outcome separately. Hence, being a smelter worker exposed to the sum of all the risk factors became the leading risk factor for hand eczema and skin complaints in this group.

**Table 4:** Risk of hand eczema and skincomplaints at other locations across occupational categories

	Crude					Adjusted*		
**Occupation**	**Odds Ratio**	**[95% CI]**		**p-value**	**Odds Ratio**	**[95% CI]**		**p-value**
Firefighter	1.0				1.0			
Chimney sweeper	0.98	0.29	3.28	0.977	0.36	0.07	1.74	0.206
Smelter worker	3.72	1.26	10.93	0.017	4.36	1.31	14.43	0.016
**Skincomplaints other locations**								
**Occupation**								
Firefighter	1.0				1.0			
Chimney sweeper	0.78	0.33	1.85	0.571	0.69	0.28	1.73	0.432
Smelter worker	2.22	0.97	5.08	0.058	2.25	0.98	5.18	0.057

*Adjusted for sex and chilhood eczema

## Discussion

In this study we found that smelter workers had a lifetime prevalence of 28.3% for hand eczema. This is twice as high as that found in the general population in a Norwegian study that is comparable to our study population [14%; 95% CI:12.6–16.5%] [10]. The point prevalence was more than twice as high as the pooled estimate in the general population in the same study population [10 vs. 4.0%; 95% CI: 2.6–5.7] [10]. Another study found a population lifetime prevalence and point prevalence of 11 and 3.4%, respectively [7].

Smelter workers had an over four-fold odds-ratio of hand eczema and an almost two and a half-fold increased odds-ratio of skin complaints at other locations compared to the other occupational groups of our study. However, it was impossible to relate this increased risk of skin complaints to any specific risk factor to which smelters are exposed; instead, the results indicate that all the risk factors combined (showering, hand wash, heat, gloves, clothing, particulate matter exposure etc.) are important and that working at a smelter with all exposure factors combined, increases the risk of hand eczema. Even though we cannot conclude any causality from our cross-sectional data, there are several other data strengthening the suspicion that the higher lifetime and point-prevalence of skin complaints in smelter workers is due to factors at work: Smelter workers were the group where most participants reported onset of eczema while working in the occupation, worsening of skin complaints at work and improvement away from work. It was the only group that had sick leave due to hand eczema.

In contrast to smelter workers, chimney sweepers and firefighters had a lifetime prevalence corresponding to the general population in the above-named Norwegian study [10]. Point prevalence in firefighters and chimney sweepers was half that of the general population [1.9 and 1.4 vs. 4.0%] [6]. Thus, the lifetime and point prevalence in firefighters and chimney sweepers in our study corresponded to that in the general population, and it seemed they did not have a higher or lower risk for hand eczema.

Skin examination revealed more visible skin symptoms and signs in smelter workers. This indicates more skin problems in this group, compared to the other groups. We have not distinguished between allergic and irritant contact dermatitis in this paper, mainly because distinguishing the two clinically are almost impossible, and we had no access to patch testing, except for in one participant. The focus was assessing differences in self-reported skin complaints between the occupational industries, which this study indicates there is.

Particulate matter generated in smelter plants and during firefighting contain high concentrations of UFP, which contain different metals, including nickel (Ni), zinc (Zn), lead (Pb), cadmium (Cd), cobalt (Co), magnesium (Mn), iron (Fe), and silica (Si) [15–17, 32, 34–37, 44]. Chimney sweeping also generates particulate matter of ultrafine fraction (PM0.1) [40], but mainly larger carbon (soot) particles. The concentrations are much lower than the particles generated during smelting or fires [39, 40]. Fine and UFP may penetrate clothing and, thus, the skin [23–25]. The skin can also be sensitised to these metals and contact allergy can occur anywhere on the skin [23, 24]. But we do not know if these particles also can act as irritant.

Smelter workers averaged twice the amount of time spent in particle-exposed areas compared with that by chimney sweepers (4.1 vs. 2.3 h/day); firefighters in this study were not exposed to ultra fine particles from fires daily. Chimney sweepers reported particle exposure not only when sweeping but also during vehicle transport from base. We joined chimney sweepers during some of their normal workdays, and an additional study was conducted where their UFP concentrations was measured across a workday during both normal and “high-exposed” week [40]. This study reported an eight hour time weighted average ultrafine (TWA) particle exposure time ranging from 2.0 × 10^3^- 9.6 × 10^3^ particles/cm^3^, while a study on our smelter workers found a TWA ranging from 1.47 × 10^4^-2.08 × 10^4^ particles/cm^3^ demonstrating a much higher exposure of UFP for smelter workers than chimney sweepers [32].

There were no differences in hand washing frequencies between the groups, despite sampling comprising time periods before and during the Coronavirus disease 2019 (COVID-19) pandemic. Sampling of firefighters was conducted during the COVID 19-pandemic, and chimney sweepers from November 2022 to June 2022. Despite being sampled before the COVID-19 pandemic, smelter workers had the highest lifetime and point prevalence of hand eczema. There was no information regarding the use of alcohol rubs for hand hygiene. However, during sampling, we found that smelter workers used soap containing microgranules to scrub the skin during hand washing, which further increases mechanical stress on skin and thus may increase their risk of having hand eczema.

Smelter workers reported almost 50% longer mean daily use of protective gloves than the chimney sweepers but reported using primarily leather and fabric gloves. Chimney sweepers reported using airtight rubber gloves underneath fabric gloves. Airtight rubber gloves are associated with a higher risk of hand eczema [5]. However, by observation, the chimney sweepers often changed the rubber gloves between sweeps, giving the skin time to dry from the moisture. Smelter workers reported changing their gloves very seldom, which furthermore may act as a source of particle exposure to their hands because of particle entrapment. Furthermore, two-thirds of the smelter workers reported exposure to heat for over 2 h per day, almost eight times the number of hours exposed for chimney sweepers. Heat in combination with occlusion, such as gloves or clothes may precipitate eczema [3] and may also be a source to why smelters had a higher lifetime and point prevalence. The mediator analysis on glove use, years in occupation and such did not change the OR significantly. This might be due to a healthy worker effect, where workers with hand eczema elicited or worsened by the job, quit their job. Workers with previous hand eczema will most likely experience a worsening of their symptoms after a shorter time of exposure, and therefore quit earlier.

To the best of our knowledge, the assessment of work-related skin complaints in locations other than the hands, is yet to be reported. In this study, there was a good correspondence between the locations of self-reported skin complaints and where participants marked where they were most visibly dirty from particle exposure (Fig. 1). Despite there not being a statistically significant difference between the occupational groups, there is an indication of a higher prevalence among smelter workers (*p* = 0.057). All groups most frequently marked skin areas not covered by garments, such as the wrists, face, head, and neck. Smelter workers, however, also reported noticeable skin complaints from their shins and behind the knee, with itching and dry, flaky skin as symptoms. They used thin wool as a base layer on their body and coarse wool on their feet throughout the work shift, with occlusive protective shoes covering parts of their legs. Wool can influence the wearer’s perception of garment tolerance, and long fibre-ends coarser than 30–32 μm are more prone to activate itch-neurones [45]. Occlusion, sweat and heat, coarse wool, particle deposition on smelter workers’ skin may give rise to skin complaints, such as itching, as the smelter workers reported on their shins. In all the occupations, thin wool was used underneath their outer protective clothing layer. Chimney sweepers used different types of outer layer clothing depending on their location. For instance, some stations used the same types of outer layers as the firefighters, while some used a thin polyester suit with buttons.

Skin complaints on the head, face, and neck was similar between the groups. This indicates that skin complaints at these locations are not necessarily related to occupational exposure to particulate matter but correspond to the typical locations of common skin diseases such as atopic eczema, rosacea, seborrheic eczema, and psoriasis. This is strengthened by the fact that we did not find any association between skin complaints and years in occupation. This might be due to a possible healthy worker effect, where those with skin complaints change occupation. Moreover, common skin conditions may be worsened by occupational exposures, such as heat, sweat, cold, prolonged use of respiratory protective equipment, particle exposure, or a combination of these [46–49]. Nonetheless, it is essential to prevent worsening of any skin condition, as this may also be bothersome for workers.

### Strengths and limitations

Although we used questions from the NOSQ-2002, we extended the questionnaire with questions to cover skin complaints at other locations. These are not validated but derived from the hand eczema questions. However, the question was formulated in a way that could confuse participants. The questionnaire asked, “Do you have skin complaints other than eczema or urticaria”? A complete figure drawing was then presented to the respondents, in which they could mark the areas of their skin complaints. A more precise question would have been, “Do you have eczema, urticaria or other skin problems at locations other than your hands?” This could mean that the question had an interpretation bias that was dependent on the occupational group. Participants with eczema at other locations may have answered “no”, thus underestimating the prevalence of skin complaints at other locations. Recall bias may be present as workers with current eczema could be more inclined to remember earlier periods with eczema and may also have had a better awareness of risk factors.

The smelter worker group is more heterogeneous regarding their work tasks than chimney sweepers and firefighters. The smelter group comprised electricians, mechanics, oven workers, tappers, energy recovery workers, and storage workers. This implies that there could be different degrees and exposure types at the individual level, which could influence the results. However, the groups were too small to conduct stratified statistical analysis.

Chimney sweepers were recruited from different parts of Norway, which increased the external validity of the findings for this occupational group. In contrast, firefighters and smelter workers were only recruited from one location and type of smelter plant, which limits the generalisation to these or similar settings.

Our study only comprised 186 participants, a relatively low number. In addition, only a small portion of workers were clinically examined, which also is a limitation. On the other hand, the response rate was close to 100% in firefighters and chimney sweepers and two-thirds in smelter workers. Even though the participation rate was lower among smelter workers, we consider the participation rate satisfactory considering the general difficulty in recruiting research participants [50]. We observed genuine interest from workers and employers in participating and contributing to a healthier work environment. Physical information meetings with oral presentations and written information may have contributed to high participation rates. We cannot rule out the possibility of a selection bias associated with the participation of smelter workers. We did not know the skin status of the smelter workers who did not participate (25.6%) or why they did not want to participate. The direction in which this may have influenced the results is unknown since people with existing conditions may or may not have been drawn to participate in the study.

The likelihood ratio test depends on including the major confounders and mediators, and we have tried to identify and include as many as possible, but may have overlooked some factors, resulting in a less effective test.

To the best of our knowledge, no previous study has investigated skin complaints in these occupations to this extent. This cross-sectional study does not allow for conclusions regarding causality, but may be generalised to firefighters, chimney sweepers and smelter workers with similar work-environments. As many of our findings are new, they make a valuable contribution to occupational dermatology, especially regarding skin complaints at locations other than the hands and arms.

## Conclusion

The lifetime and point prevalence of hand eczema and whole-body skin complaints in these three industries is new knowledge, and the results indicate a higher prevalence of both hand eczema and skin complaints among smelter workers. Our data raise suspicion that skin complaints and hand eczema in smelter workers may be attributed to a range [51] of work-related risk factors. The results should be viewed as preliminary, and longitudinal studies with a larger population are needed to establish causation.

Nonetheless, reducing hand eczema risk factors is always favourable. To achieve this, a knowledge-based approach directly to workers regarding risk factors and how to avoid them could be implemented. A previous study focusing on respiratory protective equipment in the same smelter plant proved effective [51]. Advice on daily clothing- and glove change, changing soaps from scrubbing soaps to oils and implementing use of skin moisturisers and maybe also organisational changes within the workplace may be effective in the case of skin health. Further studies on intervention effectiveness of a knowledge-based intervention may be of use.

## Data Availability

Dataset is not available as this is regarded as sensitive information.
